# Physical Fitness and Exercise During the COVID-19 Pandemic: A Qualitative Enquiry

**DOI:** 10.3389/fpsyg.2020.590172

**Published:** 2020-10-29

**Authors:** Harleen Kaur, Tushar Singh, Yogesh Kumar Arya, Shalini Mittal

**Affiliations:** ^1^Freelance Researcher and Activist, Jaipur, India; ^2^Department of Psychology, Banaras Hindu University, Varanasi, India; ^3^Amity Institute of Behavioural and Allied Sciences (AIBAS), Amity University Uttar Pradesh, Lucknow, India

**Keywords:** COVID-19, physical fitness, exercise, lockdown, gym workout

## Abstract

The COVID-19 pandemic has brought this fast-moving world to a standstill. The impact of this pandemic is massive, and the only strategy to curb the rapid spread of the disease is to follow social distancing. The imposed lockdown, resulting in the closure of business activities, public places, fitness and activity centers, and overall social life, has hampered many aspects of the lives of people including routine fitness activities of fitness freaks, which has resulted in various psychological issues and serious fitness and health concerns. In the present paper, the authors aimed at understanding the unique experiences of fitness freaks during the period of lockdown due to COVID-19. The paper also intended to explore the ways in which alternate exercises and fitness activities at home helped them deal with psychological issues and physical health consequences. Semi-structured telephone interviews were conducted with 22 adults who were regularly working out in the gym before the COVID-19 pandemic but stayed at home during the nationwide lockdown. The analysis revealed that during the initial phase of lockdown, the participants had a negative situational perception and a lack of motivation for fitness exercise. They also showed psychological health concerns and overdependence on social media in spending their free time. However, there was a gradual increase in positive self-perception and motivation to overcome their dependence on gym and fitness equipment and to continue fitness exercises at home. Participants also tended to play music as a tool while working out. The regular fitness workout at home during the lockdown greatly helped them to overcome psychological issues and fitness concerns.

## Introduction

The COVID-19 pandemic is a massive global health crisis (Bavel et al., 2020) and rapidly spreading pandemic (Bentlage et al., 2020) of recent times. As compared to the earlier pandemics the world has witnessed, the current COVID-19 pandemic is now on the top of the list in terms of worldwide coverage. This is the first time the whole world is affected simultaneously and struck strongly in a very short span of time. Initially, the death rate due to COVID-19 was around 2%, which has now increased to around 4–6% (World Health Organization [WHO], 2020). The statistics does not look so severe, but the total number of cases and the rate at which these cases are increasing day by day make the situation alarming. Exponential growth in COVID-19 cases has led to the isolation of billions of people and worldwide lockdown. COVID-19 has affected the life of nearly each person around the world. The difference between personal or professional lives has narrowed due to work-from-home instructions, and people’s lives are revolving around these two due to the lockdown. People have also been pondering over a vital concern at home, i.e., the importance of their health and fitness.

Although imposing lockdown or quarantine for the population has been one of the widely used measures across the world to stop the rapid spread of COVID-19, it has severe consequences too. Recent multinational investigations have shown the negative effect of COVID-19 restrictions on social participation, life satisfaction (Ammar et al., 2020b), mental well-being, psychosocial and emotional disorders as well as on sleep quality (Xiao et al., 2020), and employment status (Ammar et al., 2020d). Announcement of a sudden lockdown of all services and activities, except few essential services, by the authorities has resulted in a radical change in the lifestyle of affected people (Jiménez-Pavón et al., 2020) and has severely impaired their mental health, which has been manifested in the form of increased anxiety, stress, and depression (Chtourou et al., 2020). The sudden changes in people’s lifestyle include, but are not limited to, physical activities and exercise. Ammar et al. (2020a) have reported that COVID-19 home confinement has resulted in a decrease in all levels of physical activities and about 28% increase in daily sitting time as well as increase in unhealthy pattern of food consumption. Similar results are also reported by other researchers (Ammar et al., 2020c; de Oliveira Neto et al., 2020) as well. Although these abrupt changes have influenced every individual, many people who were regularly following their fitness activities in gyms, or in the ground, or other places before the lockdown have been affected intensely. Closure of fitness centers and public parks has forced people to stay at home, which has disturbed their daily routines and hampered their fitness activities. While compulsion to stay at home for a long period of time poses a challenge to the continuity of physical fitness, the experience of hampered physical activities, restricted social communication, uncertainty, and helplessness leads to the emergence of psychological and physical health issues (Ammar et al., 2020a,c). Varshney et al. (2020) have found that psychological problems are occurring in adults while adjusting to the current lifestyle in accordance to the fear of contracting the COVID-19 disease. However, effective coping strategies, psychological resources, and regular physical exercise can be helpful in dealing with such health-related problems during the COVID-19 pandemic (Chtourou et al., 2020).

It is important to note that physical activities (PA) and exercise not only maintain physical and psychological health but also help our body to respond to the negative consequences of several diseases such as diabetes, hypertension, cardiovascular diseases, and respiratory diseases (Owen et al., 2010; Lavie et al., 2019; Jiménez-Pavón et al., 2020). In a recent review of 31 published studies, Bentlage et al. (2020) concluded that physical inactivity due to current pandemic restrictions is a major public health issue that is a prominent risk factor for decreased life expectancy and many physical health problems (Jurak et al., 2020). Exercise is shown to keep other physical functions (respiratory, circulatory, muscular, nervous, and skeletal systems) intact and supports other systems (endocrine, digestive, immune, or renal systems) that are important in fighting any known or unknown threat to our body (Lavie et al., 2019; Jiménez-Pavón et al., 2020).

Regular physical activity, while taking other precautions, is also considered effective in dealing with the health outcomes of the COVID-19 pandemic (Chen et al., 2020). Researchers from the University of Virginia Health System (Yan and Spaulding, 2020) suggests that regular exercise might significantly reduce the risk of acute respiratory distress syndrome, which is one of the main causes of death in COVID-19 patients. Exercise and physical activities have important functions for individuals’ psychological well-being as well (Stathi et al., 2002; Lehnert et al., 2012). There is sufficient literature to show that exercise can play a vital role in the promotion of positive mental health and well-being (e.g., Mazyarkin et al., 2019). However, when health promotion activities such as sports and regular gym exercises are not available in this pandemic situation, it is very difficult for individuals to meet the general WHO guidelines (150 min moderate to mild PA or 75 min intensive PA per week or combination of both) (cf. Bentlage et al., 2020). Amidst this pandemic-related restriction (home confinements and closed gyms, parks, and fitness centers), how people cope up and find ways to continue their physical fitness remains an important question.

### Rationale for the Present Research

Since the onset of this disease, people have been confined to their homes, which has not only resulted in various psychological health issues but also challenged their physical fitness and health (Ammar et al., 2020a,b,c,d; Chtourou et al., 2020; Xiao et al., 2020). Although this pandemic situation has led to the unexpected cessation of almost all the outside routine activities of all the individuals, it has profoundly hampered the physical activities of fitness freaks (those who regularly go to the gym for their physical fitness), as gyms and other such places have been shut down due to the lockdown. However, studies addressing the issues of fitness freaks, who used to spend a significant amount of time for regular workout in order to maintain their physical fitness, health, and appearance, seem to have found no place so far in the literature in relation to the current pandemic situation. Supposedly, the unique experiences of such people, their health issues, and the ways in which they have dealt with these issues during the COVID-19 pandemic have remained underexplored.

Also, it is well-known that the COVID-19 pandemic has made it difficult for people to adequately maintain their normal physical activity patterns at home (Ammar et al., 2020a). There are plenty of studies that have addressed the impact of COVID-19 on physical activities of the general public (Ammar et al., 2020a,b,c,d; Chtourou et al., 2020; Xiao et al., 2020), demonstrated the significant decrease in physical activities and exercise patterns, and illustrated its ill effects on physical and mental health status. There is also a growing body of literature that suggests strategies to encourage people to be involved in home-based exercises and fitness activities (Ammar et al., 2020a,b,c,d; Chtourou et al., 2020; de Oliveira Neto et al., 2020). However, all these studies were conducted in the earlier phase of the pandemic. There is a lack of studies investigating the way in which people have dealt with the problems arising from the COVID-19 pandemic and subsequent lockdown/home confinement. In fact, it would be interesting to explore how and to what extent people were able to follow and benefited from the workout at home advices. Therefore, the present research aims at understanding people’s unique experiences during the period of lockdown due to COVID-19 and exploring the ways in which regular exercise engagements helped them deal with the psychological and physical consequences of home confinement.

## Methods

In order to gain a rich and extensive understanding of experiences into people’s lives during this pandemic and their efforts to maintain a healthy lifestyle, a qualitative approach was adopted for the study. We used Interpretive Phenomenological Analysis (IPA) to delve into the participants’ perceptions and to provide a close picture of the participants’ unique experiences during the lockdown period.

### Participants

A homogeneous sample of 22 participants was selected for this study. The criterion-based purposive sampling technique was used to identify and select the participants. We first contacted the gym owners/trainers and sought their consent to help us in the conduction of this study. Upon consent, we requested them to provide us with the details of their regular gym members who continuously go to the gym and do fitness exercises for at least 6 months prior to the imposed lockdown. Once the list was generated, the prospective participants were then connected by phone, were explained the purpose of the study, and were requested for their consent to participate. Those who consented for their inclusion in the study were then asked some questions based on the pre-decided inclusion and exclusion criteria for the study. On the basis of this information, those participants who met the inclusion criteria (i.e., those who were continuing fitness workout in their home or hostels and were following strict home confinement measures during the COVID-19 pandemic and subsequent lockdown) were further contacted and requested to provide an appointment for a telephone interview.

### Inclusion and Exclusion Criteria for the Participants

The participants meeting the following criteria were included in the study:

• Individuals aged 18 years or older.
• Individuals with no known history of physical and/or psychological illness.
• Individuals who were doing regular gym workout for the last 6 months or more for at least 45 min daily before COVID-19.
• Individuals who were completely dependent on gym exercise for their physical fitness.

However, individuals meeting the following criteria were not included in the study:

• Individuals who were irregular or occasional gym visitors.
• Individuals who were practicing other physical exercises besides gym workout.
• Individuals with any physical and/or psychological conditions or individuals on any kind of medication.

Table 1 presents the demographic and exercise characteristics of the participants included in this study.

**TABLE 1:** Demographic characteristics of the participants.

Variables	Variable levels	Characteristics
Gender	Male	20
	Female	2
Age (in years)	Minimum	19
	Maximum	34
	Mean age	26.5
Occupation	Student	6
	Homemakers	1
	Working professionals	11
	Entrepreneur	2
	Unemployed	4
Marital status	Single	19
	Married	2
	Separated	1
Living status	Living alone	7
	Living with family	15
Socioeconomic status	Middle class	18
	Higher class	4

### Procedure

The purpose, importance, and relevance of the study were explained to the participants, and informed consent was obtained for their participation. All the participants were assured of the confidentiality of their responses and identity. Upon consent, the participants were requested to share their convenient time for a telephone interview. Semi-structured telephone interviews were conducted to explore the exclusive experiences of the participants with regard to their physical fitness during the lockdown. An interview schedule composed of non-directive, open-ended questions was prepared. There was no fixed order of questions; they were modified and re-modified as per the flow of the conversation with each participant. Some of the main questions prepared for the semi-structured interviews included “What is your perception of this situation we are currently living in?,” “What is your lockdown experience?,” “How frequently you used to go to gym for exercise before the lockdown was imposed?,” “How do you manage exercise at home?,” “What is your exercise schedule now?,” “What changes did you perceive in yourself during this lockdown?,” “How are you coping with this lockdown?,” “Did you experience any psychological issue during this period of time?,” “How do physical exercises help in combating the crisis you are facing?,” “What background aid do you use while exercising at home?,” “What is the need to use such aids while exercising?,” “How does fatigue impact you when you exercise during the lockdown?,” “What is the importance of proper sleep in following a regular schedule of exercise during this lockdown?,” “Do you miss your gym mates?,” “Do you feel you share an identity with your fellow gym mates?,” etc. Additional probing questions were also added as the need occurred during the individual interviews. In addition questions were also asked t o understand the differences between their pre and during COVID-19 lockdown fitness exercise patterns (see Table 2). All the interviews were conducted in the native language of the participants in Hindi and English. With due permission from the participants, the interviews were recorded. The interview time duration range was between 20 and 30 min. All the interviews conducted in Hindi were transcribed and then translated in English by the researchers. The translated interviews were then proofread by a native English speaker for correctness and consistency.

**TABLE 2:** Pre- and during COVID fitness exercise information of the participants.

Fitness routine	Levels	Before COVID	During lockdown March 25th to May 30th, 2020	Lost lockdown (limited restrictions, complete gym closure) June 1st to July 31st, 2020
Variables	Frequency	Daily	3–4 days a week	5–6 days a week.
	Hours (Daily)	1–3.5 h	1–1.5 h	30 min to 1 h
	Type of exercise	Endurance training, strength training, static stretching	High-intensity workout with available equipment or substitutes at home, rope jumping, yoga	Yoga, meditation, walking, jogging, High-intensity workout with available equipment or substitutes at home
	Place of exercise	Gym	Home	Home and park
	Dependence on any other physical fitness exercises	No	Yes	Yes
	Social media accounts active	WhatsApp, Facebook	Facebook, Instagram, WhatsApp, YouTube, IMO, Telegram, Hangouts	Twitter, Facebook, Instagram, WhatsApp, YouTube, IMO, Telegram, Hangouts

## Analysis and Results

All the recorded interviews were transcribed. These transcripts were then analyzed using the Interpretative Phenomenological Analysis (IPA) framework to identify the participants’ experiences of lockdown, their alternative choice to continue their fitness routine, and its impact on their health. A stepwise progression method was used to analyze the data. At first, the researchers read the transcripts many times to get a deeper understanding of the experiences as described by the participants. In order to gain as close an understanding of the data as possible, the researchers listened to the audio recordings of the participants while reading the transcribed data.

In the following step, the attempts were made to transform the transcripts into a conceptual framework that was deeply connected to the participant’s original verbatim in order to identify emergent themes (see Table 3).

**TABLE 3:** Major themes and subthemes that emerged from the interviews indicating participants’ experiences during the COVID-19 pandemic.

Major themes
**Psychological health issues**
Having frustration, stress, anxiety, and fear
A trend of laziness and mental fatigue
Change in sleeping pattern
**Lack of motivation for fitness**
The role of gym mates
The role of gym environment
**Change of perception**
Negative situational perception
Positive self-perception
**Shifting focus on substitutes of gym workout and equipment**
Shift on yoga and meditation
Shift on high-intensity workouts
Shift on alternatives of heavy weights
**Social media dependence**
As a medium to get updated
To overcome the monotonous daily schedule
Increases the amount of sitting
Lack of emotional attachment
Platform to know virtual fitness techniques and influencers
**Favorable attitude toward music as a tool**
Used to focus on exercises
An aid that provides distraction from home setting
Creates one’s own world, where there is no COVID-19

After identifying the emerging themes, the transcripts were read again so as to cluster these emergent themes together according to their similarities at the basic level. In this process, some themes emerged as the broad themes under which subthemes were incorporated. The major themes and subthemes that emerged in the analysis are presented in Table 3.

Table 3 presents six major themes describing the experiences of participants with regard to the COVID-19 pandemic and their efforts to maintain a healthy lifestyle. The following section discusses each of these themes and its subthemes along with the relevant excerpts from participants’ experiences.

### Psychological Health Issues

Almost every participant reported facing psychological health issues linked to the COVID-19 pandemic and subsequent lockdown. Participants experienced frustration, anxiety, fear, and stress. For example, participant 11 reported,

“I am experiencing frustration daily for spending my 24 by 7 time at home, looking at same faces and am not allowed to go anywhere. Anxiety of work and its upcoming scenarios tickle my mind a lot. What if I have to do my job virtually for a lifetime? ………….Like that. And especially experiencing a fear of losing my ever charming personality, the economic status of family, no wages or less wages, fewer opportunities in future, job shift, health care of my family.”

The closure due to the pandemic has created a state of uncertainty about an individual’s own future as well as about the future of the family and community, which in turn is being reflected in terms of psychological states of frustration, anxiety, fear, and stress.

Individuals stuck at their homes without a clearly defined routine and work are not able to prioritize their work schedules, resulting in the experience of unexplained laziness and fatigue. Participant 7, for example, reports that

“Physical fatigue has reduced as there is no physical load or fixed working hours, but the mental fatigue and mental pressure has increased manifolds. Worries have increased. Spare time is more than what was required and due to this lethargy has increased. Frustration level is going up.”

The monotonous and closed life cycle of one confined to one’s own home has also resulted in extreme disturbances of one’s sleep cycle. For example, Participant 5 reports,

“Sleep a lot, a lot!! Just imagine I have been sleeping 10 to 12 hours after the lockdown. My sleep pattern was set earlier due to office, but it is disturbed now in the absence of a routine. I have virtual meetings now also, but if the meeting is to start at 10, I would get up at 9.40, wash my face and attend the meeting. After that I feel like taking a nap again. I sleep for 8 hours wake up and exercise in the morning, but I have the liberty to be flexible with my time. seriously I am craving for gyms to open, my trainer to keep a check on me, scold me, I really want complete sleep and a routine.”

It is therefore evident from these examples that the onset of the COVID-19 pandemic has resulted in the experience of psychological problems characterized by frustration, anxiety, fear, and stress. The sleep–wake cycle is interrupted, leading to a state of laziness and mental fatigue.

### Lack of Motivation for Fitness

The closure of gyms and other fitness activity centers, including sports stadiums, morning walk parks, etc., and the heightened psychological health issues have resulted in the lack of fitness motivation. For example, participant 1 reports,

“See, ultimately due to the shutdown of gym during this pandemic, my rhythm has been disturbed, you are getting it? I have had a tight schedule always due to my profession but each evening I used to hit the gym daily…………. I mean, that zeal is gone, ……….now also I am getting time in the evening but then also I am unable to ask myself to work out because that gym environment is gone, the gym people as you would see other fellows at gym, that would motivate you, their body gives you an inspiration that how he or she is that fit, they motivate you, here I share an identity with them, I find those people as source of my motivation to physical exercise, those people give you so much morale and now that is lost totally, I literally crave for that.”

The motivation for fitness is not only internal but also external. People are motivated when they observe others doing fitness activities. Gym mates and their physique work as motivating factors for individuals to engage in a regular and routine gym activity. Participant 10 said in frustration that,

“Almost all gone, ………….the motivation is the most ruined thing today, ……….talking about my workout, I have been hitting the gym since I was 22………, Imagine how much that space motivated me, I miss that, my pals there……., not because we are friends or something, see gym doesn’t provide you an environment to make pals or something as people change their gyms and many a thing but, they give you a lot of competition, you become jealous of their appearance and later that workout that space becomes your habit, I miss that, say like anything, but still I am trying.”

It is evident from the above statement that a lack of motivation for fitness was due to the home confinement and lack of presence of others. The presence of others engaged in a similar activity not only creates a sense of shared identity but also is a source of healthy competition and thus motivation.

### Change of Perception

As the days progressed, individuals learned to respond to the pandemic in a more constructive and positive manner. Their perception for the situation remained the same (negative), but their perception toward themselves started to change. They started believing that even though they could not change the situation, they could do the same for their own self to deal with the situation. Participant 2, for example, commented on the situation and said,

“Ah! Talking about the situation we are living in, it is so unprecedented, anything can happen anytime, though I am less stressed as compared to the date the lockdown was announced, I perceive this whole situation is so terrible, worst… what is this happening, you just tell me, wake up in fear and sleep in fear. I wonder when this is going to end.”

However, upon asking about her/his own self, s/he added

“You know this COVID has done only one thing right, that is, you know giving me immense time to work on myself, which otherwise I always overlooked. Though I went to gym for my physique only but never gave time to my thoughts, skills, etc. So when talking about changes in myself or perception of self, I would say changes come under three categories in me- first physical, that is appearance, personal, like I will quote enjoying every bit of time. Who knows I am next. I now celebrate life, and finally social changes in myself, as I have got time to work on my communication skills, talking on virtual platforms and sense of oneness or say unity, as I am locked down in hostel and we guys do every deed and task on our own without family, standing together.”

Similarly participant 22 summarized the situation as

“(Laughing), Seriously! The Virus is making a joke on us, truly this is the worst of situations I can ever imagine, I am so negative about the situation we are in, I am in… everyone in….you know how stressful it is for me to know that I am unable to practise. You know as a clinician how hard it is to be like this. Though I am still a student but think likewise, harsh situation madam, extra precautions for everything, negative, too much negative. This time would be a memorable time for generations; sorry my tone has become louder I am kind of in agony, all credits to this so called CORONA.”

S/he, however, further commented that

“my experience throughout the past few months in this Corona Era is so negative but myself-perception or I would say how I am taking myself now from earlier has meaningfully changed now. You know, I am someone who is giving time to myself, exploring my hobbies, giving time to leisure, learning kitchen skills, learning new dishes, becoming a chef besides being a dentist you know. So, for me, myself, I am so positive with regards to myself.”

It is therefore evident that increased experiences with an initial unfamiliar situation initiate the coping mechanisms within an individual, which is reflected in the changed perception of their own self, and reappraisal of the situation in a more positive manner.

### Shifting Focus on Substitutes of Gym Workout and Equipment

With the positive change in perception, individuals started to think about their normal routine and tried to find ways to substitute their normal activities. They started trying to shift their exercises from gym to other available places and using alternatives to gym equipment for their fitness activities. The statement of participant 20 indicated how shifting from gym-based exercises to yoga practices was an effective alternative for coping with the habitual compulsion for gym exercises.

“Since I get a pace back again for my physical fitness in this lockdown, I have made a shift to yoga, especially the power yoga in the morning time. I prefer doing meditation as well. Earlier I never used to practise the same but now I have seen videos of some asanas good for health, I am following them and practising them. It’s a shift for peace I guess. I tried something new and found my gym addiction could be controlled or moderated by taking out time for yoga and meditation even after COVID.”

Similarly, participant 17 reported her/his shift to high-intensity workouts at home.

“See, as you might know not everyone has exercise equipment at home which we used to have in gym. So, I prefer those exercises which require less or zero weights say jumping jacks, skipping.”

After resuming motivation, in order to stay physically active and fit, participants actively engaged in the process of finding alternatives to their routine physical exercise equipment. Participant 14 reported shifting to alternatives to heavy weights

“I personally was too much dependent on equipment to exercise in the gym. Now there is no option left because even online, the 5 and 10 kg weights are out of stock, And, nearby stores are either closed or you can’t go out. So, for me it was tough but I searched the internet, the social media, talked to fitness experts and used some ‘JUGAAD’ at home. So, they are using buckets, big water bottles and skipping ropes. I had 10 kg iron rods of water pipeline spare at my home, I am using that and these are helpful and I guess need of the hour.”

### Social Media Dependence

One of the major shifts in the individuals’ lives during this pandemic was the increased social media dependence. As a result of social distancing, people were spending more time online to virtually connect with others and stream entertainment. In the backdrop, the COVID-19 pandemic led to an increase in the time spent on social media that helped people kill time. Participant 12 reported the benefits as well as the drawbacks of this social media dependence.

“Social-media is a mixed feeling platform. I mean at one hand it keeps me updated with the happening around; the facilities promised by the government; and… it keeps me connected with the world. But on the other, it irritates me a lot, a lot of misinformation creates a worry in you. So yes, there is a dual objective of this social media.”

However, participant 4 viewed this increased dependence on social media as an effective strategy to break the silence and to overcome the monotonous days.

“Our life has given us so much time ……., I mean I have so much spare time but besides that, I have a monotonous schedule every day, so social media keeps me busy, for example, web series suggestion and reviews, movies suggestion and reviews, video games, etc. Also, on the one hand, I do not get bored as one day I am learning some planting technique at home through media, the other day something to cook, some family or friend sharing his/her recipe, hobby ideas, craft ideas, writing, etc. Physical workout schedule helps me a lot. I am doing one thing useful at a time, and that keeps me busy.”

Similarly, participant 3 reported that

“Definitely social media has impacted my sitting schedule as I am just sitting for a long span of time, say while eating or talking to family. I am sitting scrolling YouTube, Facebook, WhatsApp, Instagram, one post after the other. It has become my habit now. I feel like I will only watch a single video or only this news but I end up spending 1 to 2 hours scrolling and watching. Seriously, it’s a habit now, but I am glad that workout is something I do in my schedule, which is so productive, and I really feel good about myself because of the physical fitness.”

However, participant 21 pointed out the experience of lack of emotional attachment, sympathy, and support resulting from the content consistently served by social media.

“Social media is full of content which reveal crime stories, life matters, relationships, suicides, etc. at a large scale. So many movie clips, videos, web series show a lot of crime, aggression or say anything on that. So, I feel now-a-days emotionally detached to any relationship, friendship or even to my family. If I receive their call, I would say yes okay fine, no further interest in how they are dealing or what they are experiencing. And if they ask I would say, so what, I am not a kid anymore. I lead my life you lead yours, definitely social media is making me someone I never used to be. In fact, my sister has become the same, though she is living with the family under the same roof. Earlier I was so sensitive to any suicide or crime. If I heard of that I would cry or be sad. I used to feel the pain of the victim. Now, I hear a story for real and I am like, yeah part of life, or you pay for deeds like that. No sympathy left I guess, so detached.”

However, what was more important was that social media was seen to be helping individuals in maintaining their daily fitness routines by providing them alternative fitness tools and techniques, the virtual company of other fitness freaks, and by helping them back, influencing others and getting influenced by others. Participant 6 reported that

“Social media has lots of side effects, but a good effect of it now-a-days for a gym freak like me is that social media provides videos of trainers, and other freaks working out at home or hostels. I can know now virtually how to maintain a schedule. They are sharing their experience, they are influencing me a lot, I am trying my best, and workout is helping me a lot.”

### Favorable Attitude Toward Music as a Tool

Many participants also reported the use of music as an aid while exercising. Participant 7 reported that

“I have two schedules of exercise. If working out in the morning, I prefer soothing music, like that of birds chirping, or instrumental jazz. And if I am exercising in evening, I want to listen to EDM, that is electronic dance music, I have made a playlist of computerised music and listen to that in evening. I prefer music because it takes you to another world, which is needed the most now (exclaimed!) It creates an environment like that of a gym in my head, or say, I imagine I am in the gym, as I cut off all the surrounding voices.”

Similarly, participant 9 reported that

“I just love to have old-country music while I am exercising. It is a kind of genre of songs, the old country one, and sometimes I love random numbers of songs. It is needed because you can say it lets me focus, helps me to calm down. Also, when I am locked at home, it actually provides me a world free of distractions, just my own world, where there is no corona. Music is ultimate fun. If there is no music available I will not workout, because workout makes me happy and I really want to exercise effectively and enjoy it too.”

It is, therefore, evident that music is an important supporting tool that helps individuals relax and enjoy their original routine even when they are working out at home. Music is a powerful tool that recreates the same environment that participants used to have during their gym exercise times.

## Discussion

The COVID-19 pandemic has brought major upheaval in the life of every individual across the globe. It has hampered the day-to-day activities of almost all individuals including those who depend on gyms for their physical fitness routine. The present study was conducted with individuals for whom going to the gym was a routine activity so as to explore their experiences in terms of their perceptions of the pandemic situation and their ways of coping with COVID-19-induced uncertainties and health issues.

The findings of this study not only are consistent with a range of studies that have reported psychological health issues due to the COVID-19 pandemic and subsequent lockdown (Hawryluck et al., 2004; Ammar et al., 2020a,b,c,d; Chtourou et al., 2020; de Oliveira Neto et al., 2020; Shigemura et al., 2020; Varshney et al., 2020) but also go beyond those to suggest that, with time, individuals learn to adopt to situations in healthy and positive ways. Participants reported experiencing a significant change in their sleeping pattern, unexplained laziness, and mental fatigue, and having a general feeling of fear, anxiety, stress, and frustration due to home confinement, which impacted their motivation to find alternate ways to continue fitness exercises.

Other factors found responsible for the lack of fitness motivation were the absence of gym partners and the lack of gym environment, which were also considered as potential sources of gym motivation in earlier studies (Sonstroem and Morgan, 1989; Sonstroem and Harlow, 1994; McAuley et al., 2000; Fox, 2003; Tamur, 2014). It is important to note that, being a social entity, people like the company of others and feel connected to each other. This feeling of connectedness is found to be associated with various psychological constructs such as persistence, motivation, self-esteem, self-efficacy, and physical as well as psychological health (Scully et al., 1998; Proctor et al., 2011; Haslam et al., 2015; Begun et al., 2018). The absence of this feeling of connectedness that people were used to experiencing in a gym environment probably was one of the reasons for the lack of motivation for home exercise.

The findings of the study also indicated that although the participants’ perception of the pandemic situation was negative initially, their self-perception gradually improved toward a positive one, as they realized that they had enough time to look after themselves. Rauthmann et al. (2015) reported that environment and behavior, if different from the usual, lead to a negative situational perception. However, with an increase in time available to devote to oneself, perceptions change in a positive direction (Karagiannidis et al., 2015). Such a change in perception is likely to promote the process of self-approval and find effective ways to deal with the current situation.

In the present study, a shift from the gym workout and fitness equipment toward substitutes is clearly visible during the latter part of the lockdown. After the initial confusion and passive wait for things to normalize, participants accepted the reality and started thinking about alternatives to exercises related to heavy gym equipment. Some of the alternatives listed by them included switching to yoga and meditation (National Center for Complementary and Integrative Health, 2020), high-intensity workout at home, and lifting heavy buckets, big water bottles, and skipping. All these alternative arrangements not only helped individuals maintain their daily exercise routine but also contributed to their physical and mental health (Jiménez-Pavón et al., 2020; Nicol et al., 2020). In fact, the American College of Sports Medicine had recommended 150–300 min of aerobic exercise per week and two sessions per week of moderate-intensity muscle strength exercises for people to be physically active during the COVID-19 pandemic (Joy, 2020).

The mixed impact of social media usage and listening to music during exercise was also observed in this study. Results clearly indicate that participants found social media to be an effective medium to keep themselves up to date about the pandemic situation and to overcome the monotony of home confinement. Apart from this, participants also experienced a lack of emotional attachment, as face-to-face interaction during the said period was missing. This encouraged participants to use social media to get connected to people as well as to witness their regular activities, which they were missing otherwise. Several studies in the past have argued that social support boosts motivation for training and can increase up to 35% more adherence to a physical exercise program (Rhodes et al., 2001) and that it can be an additional strategy to make exercise events more interactive and less dissociated from afferent body responses (heart rate, breathing), which in turn results in more positive training experience (Kravitz and Furst, 1991; Pridgeon and Grogan, 2012).

Social media was also used as a platform to know about virtual fitness techniques and opportunities for online training for physical exercise. Ammar et al. (2020d) demonstrated 15% higher use of Information and Communications Technology (ICT) during the COVID-19 confinement duration, which indicates higher use of social media and app use for home-based fitness activities (Tate et al., 2015; Ammar et al., 2020a).

Furthermore, participants also found that listening to music was an effective aid to keep themselves engaged as they exercised. This also supports the finding that music helps people to continue their fitness workout for a significantly longer period of time (Thakare et al., 2017). A series of studies have shown that music creates an ergogenic effect during physical and cognitive performance and is linked to heightened motivation and engagement and lower levels of stress, anxiety, and depression (Chtourou et al., 2015). In their recent meta-analytic review Terry et al. (2020) have concluded that listening to music during physical activity boosts positive affective valence and results in improved physical engagement and enhanced physiological responses. It is therefore clearly evident that listening to music while doing physical exercise during the current pandemic has enabled people to focus on the exercise without any distraction from the home setting and has enabled them to create their own world, where there is no COVID-19.

To conclude, the findings of the study indicate that the perceptions and social media habits of fitness freaks, who were hitting gyms for a regular workout before the lockdown, were greatly impacted by the COVID-19 pandemic. They also experienced psychological health issues during the initial phase of the pandemic. However, they gradually changed their dependence on gym-based workout and switched to alternative exercises that helped them greatly to restore their mental and physical health.

### Implications and Future Suggestions

The present study shows that despite the initial experience of anxiety and fear and the lack of motivation to engage in physical exercise at home, fitness freaks were able to shift to home exercises and were greatly supported by social media uses and listening to music. One could argue that this study only included fitness freaks who find it difficult to detach themselves from physical activities for a long time, and this was probably the reason for their shift to home-based exercises. However, there is no doubt that the findings of this study have demonstrated that if performed regularly, physical exercise has the potential to mitigate the ill physical as well as psychological effects of the COVID-19 pandemic. The findings of this study, therefore, could be extended to the common public to also persuade them to engage in physical fitness exercises, which would result not only in a better physical health but also in an enhanced psychological health and well-being. The findings of this study strengthen the recommendations made by researchers and organizations (for details see Chtourou et al., 2020; World Health Organization [WHO], 2020) to engage in home-based exercises (including, but not limited to, aerobic activities, balance and flexibility exercises, and muscular strength and endurance training) for about 150–180 min per week; to use social media, music, and/or similar techniques to increase adherence to physical exercises; and to practice dancing and yoga to reduce stress, anxiety, and depression, and even improve the quality of sleep (Chennaoui et al., 2015; Chtourou et al., 2015). It is also noted that one should start physical exercise and its alternatives in a progressive manner and must adhere to his/her fitness levels for choosing the amount and intensity of these exercises.

## Data Availability Statement

The raw data supporting the conclusions of this article will be made available by the authors, without undue reservation, to any qualified researcher.

## Ethics Statement

All procedures followed in this study were in accordance with the APA’s ethical standards and with the Helsinki Declaration of 1964 and its later amendments. The patients/participants provided their written informed consent to participate in this study.

## Author Contributions

HK, TS, and YA conceptualized the study. HK and TS prepared study protocols. HK collected data, conducted initial data analysis, and wrote the first draft. TS, SM, and YA finalized data analysis, reviewed, and commented on the draft manuscript. HK, TS, SM, and YA contributed to the preparation of the final draft. All authors contributed to the article and approved the submitted version.

## Conflict of Interest

The authors declare that the research was conducted in the absence of any commercial or financial relationships that could be construed as a potential conflict of interest.
